# Depression among older adults in Norway 1995–2019: Time trends, correlates, and future projections in a population study: The HUNT study

**DOI:** 10.1371/journal.pone.0328413

**Published:** 2025-07-31

**Authors:** Maria Lage Barca, Eivind Aakhus, Ellen Melbye Langballe, Thomas Hansen, Ragnhild Holmberg Aunsmo, Geir Selbæk, Steinar Krokstad, Bjørn Heine Strand

**Affiliations:** 1 The Norwegian National Centre for Ageing and Health, Vestfold Hospital Trust, Tønsberg, Norway; 2 Department of Geriatric Medicine, Oslo University Hospital, Oslo, Norway; 3 Old Age Psychiatry outpatient clinic, Oslo University Hospital, Oslo, Norway; 4 Department for Mental Health and Suicide, Norwegian Institute of Public Health, Oslo, Norway; 5 Promenta Research Center, University of Oslo, Oslo, Norway; 6 Verdal municipality, Norway; 7 Institute for Clinical Medicine, University of Oslo, Oslo, Norway; 8 HUNT Research Centre, Department of Public Health and Nursing, Faculty of Medicine and Health Sciences, Norwegian University of Science and Technology, NTNU, Norway; 9 Levanger Hospital, Nord-Trøndelag Hospital Trust, Levanger, Norway; 10 Department for Physical Health and Aging, Norwegian Institute of Public Health, Oslo, Norway; Tampere University, FINLAND

## Abstract

**Objectives:**

To investigate patterns and correlates of depression among Norwegian older adults (age 70+), 1995–2019, and estimate the number of older adults with depression by 2050.

**Design:**

Population-based cross-sectional study

**Setting and participants:**

Three surveys of the Trøndelag Health Study (Norway): HUNT2 (1995−96), HUNT3 (2007−08), and HUNT4 (2017−19). 22,822 home dwellers aged 70 + who participated in at least one of the three surveys.

**Methods:**

Depression was defined as scores ≥8 on the depression subscale of the Hospital Anxiety and Depression Scale. Covariates included sex, age, education, marital status, and reported loneliness. Depression prevalence (%) was standardized to the Norwegian population by age, sex, and education for years close to the initial HUNT survey year (1995, 2006, and 2016). Projection of the total number of individuals with depression in the coming decades were estimated. Predictors of depression were analyzed with logistic regression and the potential reduction in depression prevalence by reducing the prevalence of loneliness was estimated.

**Results:**

Standardized depression prevalence decreased from 16.7% (HUNT2) to 14.9% (HUNT3), and 11.5% (HUNT4), and was highest among men, the oldest (85+), the lower-educated, and in earlier surveys (all p < 0.001). Living alone was also associated with higher depression prevalence, but only if loneliness was present. While depression rates are falling, we expect the number of depressed individuals to double by 2050 as the population ages.

**Conclusion and implications:**

Depression rates among adults aged 70 + decreased by 50% from 1995 to 2019, but less so among the oldest old. The rates were highest among single older men. Despite decreasing prevalence, the number of depressed older adults will increase significantly in the future. Given the major individual and societal costs of depression, this trend is alarming for societies preparing for the challenges posed by population aging. This can, however, be addressed by addressing predictors of depression.

## Introduction

Depression is one of the most common psychiatric disorders among older adults, with severe adverse consequences for functioning in daily living [1], physical health [2], loneliness [3], morbidity and mortality [4], the risk of dementia [5], and suicide rates, especially among older men [6]. Not surprisingly, therefore, late-life depression also has major costs to families, local communities, and societies [7]. The prevalence of depression in the general older adult population is 10–30% [8–10], with higher rates including subclinical depression or using depression scales and lower rates with clinical diagnostic criteria [11–13]. With the growth of the older population and changes in known depression predictors like health and socioeconomic resources, it is imperative for researchers, public planners, and stakeholders to comprehend the shifting risk of late-life depression as these dynamics evolve.

In Norway, a recent study reported diverging time trends in depression according to age; there has been an upward trend among young individuals, contrasted by a decline in adults aged 50–79 years [14]. Time trend studies for the oldest old are lacking. However, another recent Norwegian study reported an elevated depression prevalence at a fixed time point among older adults over 80 years compared to younger adults [15]. This finding aligns with international results from systematic reviews [8,9]. Sex differences have been reported, revealing higher levels of depressive symptoms among older men but not women [16]. This contradicts the conventional notion of female sex as a risk factor for depression [8]. Discrepancies related to sex might also stem from methodological variations, including the definition of depression employed [14,17–19]. Additional factors, including loneliness, living alone, lower education, low socioeconomic status, cognitive impairment, and physical illness, have also been identified as contributors to an increased risk of depression [3,8]. This has become even clearer during the COVID-19 pandemic, as depression in this age group was associated with social isolation and loneliness [20,21].

The aging of the global population, projected to double the number of people over 60 years from 12% to 22% by 2050 (WHO), is a crucial determinant in the increasing number of older adults with depression. Depression is projected to be the second leading cause of disease burden by 2030. Considering the demographic shifts and the profound socioeconomic implications of depression, it is imperative for policymakers to obtain accurate insights into the evolving trends and anticipated shifts in late-life depression patterns and risks. Although a meta-analysis reveals an overall increasing trend in the likelihood of experiencing depression, heterogeneity across studies prevented the analysis of subgroups [22]. However, scant research has addressed these issues using large, high-quality data. Furthermore, projections should ideally take into account changes in the older population, for example, in terms of health, education, and social connectivity. Notably, efforts like Norway’s strategies to mitigate social isolation and loneliness in older adults are critical, as loneliness is a major contributor to depression. Effective interventions in reducing loneliness could yield significant benefits in curbing depression rates among the elderly.

The aim of this study is to examine temporal trends in the prevalence of depression, focusing on variations by age, sex, education, living situation, and reported loneliness among Norwegian adults aged 70 and older from 1995 to 2019. Additionally, we seek to project the number of older adults with depression in Norway by 2050. As an adjunct, we will investigate how these projections might be influenced by shifts in the prevalence of loneliness within this demographic.

## Materials and methods

### Study design and participants

A cross-sectional study design was applied, and the study sample was based on Norwegian regional data from the three most recent surveys of the Trøndelag Health Study (HUNT) collected during a 24-year period: HUNT2 in 1995–1997, HUNT3 in 2006–2008, and HUNT4 in 2017–2019. The study sample was home dwellers aged 70 years and older when participating in one or more of the study waves.

The northern part of the Norwegian Trøndelag County is characterized by a stable and homogeneous population. In the HUNT Study, all county residents aged 20 years and older were invited [23]. In HUNT2 (1995−97), 16,235 adults aged 70 years or older were invited, of whom 10,939 (67%) participated. In HUNT3 (2006−08), 14,982 were invited, of whom 8,412 (56%) participated. In HUNT4 (2017−19), 19,239 were invited, and 12,092 participated; of these, 512 were excluded due to being nursing home residents, leaving 11,580 (60%) for analysis. In HUNT2 and HUNT3, there was no data collection in nursing homes. Thus, out of the total 65,561 invitations to participate in HUNT2–HUNT4 issued to those aged 70 years and older, 42,379 were included. Our study sample comprised 22,822 individuals with valid values for HADS-D and 26,512 assessments. Most of these respondents (79.5%) participated in only one survey, and some participated in two (19.6%) or three (0.9%). Even if some participated in multiple surveys, each survey was treated as an independent sample of those aged 70 + . For example, a respondent aged 70 in HUNT2 (1995) would be 81 in HUNT3 (2006) and 92 in HUNT4 (2017) and would thus be analyzed as three separate individuals. First data file from HUNT delivered to the project was Oct 01 2019, and additional variables were delivered 21 April 2023. Date inclusion HUNT2: Aug 15 1995 – June 18 1997. Date inclusion HUNT4: May 05 2017 – Feb 21 2019

### Variables

#### Dependent variable.

We measured depression using the depression subscale of the Hospital Anxiety and Depression Scale (HADS-D) [24]. HADS-D comprises seven questions and is scored on a four-point Likert scale (0-1-2-3). Scale scores range from 0 to 21, with higher scores indicating greater levels of depression. The HADS instrument has been validated for older adults [25]. Previous studies have proposed various cut-off values for HADS-D [19,26]. In the present study, those with a sum of ≥8 on the HADS-D were defined as depressed, based on findings from a validation study [26]. As suggested by Bell et al., 2016 [27], for population studies, we imputed missing items using the subject’s subscale mean if at least half of the items (four or more) were answered (the so-called half-rule). For HUNT2, HUNT3, and HUNT4, the percentages imputed were 11.4%, 1.7%, and 1.5%, respectively.

#### Independent variables.

We measured loneliness by a single item: “Have you felt lonely during the last two weeks?” This question was taken from the seven-item questionnaire in the Cohort of Norway Mental Health Index (CONOR-MHI) [28]. There are four possible responses: 1) no, 2) a little, 3) a good amount, and 4) very much. Categories 3 and 4 were collapsed in our analysis, resulting in a three-point scale labeled 1) never, 2) sometimes, and 3) often. We extracted self-reported demographic variables, such as sex, age, education, and living situation, from the HUNT database. We grouped age as 70–74, 75–79, 80–84, and 85 years and over. We classified education as primary (<10 years), secondary (10–12 years), or tertiary (13 + years), and dichotomized living situation into 1) Living alone (for the response “lived alone”) or 2) Living with someone (for all other responses).

### Statistical analysis

We used Stata 17 SE (StataCorp LLC, USA) for the analyses. Five sets of numbers were estimated. First, we estimated the crude prevalence of depression by sex, age, living situation, and loneliness. Second, we standardized the prevalence of depression to the Norwegian population using three sets of Norwegian standard populations by age (70–74, 75–79, 80–84, 85+), education (primary, secondary, tertiary), and sex (men, women), one set for each HUNT survey. The years used were 1995 for HUNT2, 2006 for HUNT3, and 2016 for HUNT4 (2016 and not 2017 was used for HUNT4, as this was the latest year for which we had weights available for education). Weights (number of people in each of the 72 strata) were drawn from the web-based service microdata.no. The purpose of the standardization was to get national estimates and to correct for lower participation rates in the higher age groups, among men, and in the lower education groups. The 70 + Norwegian population size was n = 497,679, n = 491,878, and n = 576,537 in 1995, 2006, and 2016, respectively. Thus, the total weight in our study equals N = 1,566,094. Standardized prevalence of depression for men and women in each HUNT survey was estimated using the svy command in Stata, and differences between the sexes were assessed using the Fisher-test. Third, projections of the total number of individuals with depression in the coming decades were estimated, fixing the standardized prevalence of reported loneliness in HUNT4 by age and sex. These prevalence data were then multiplied by population data from Statistics Norway by the same age groups and sex for the years 2020, 2025, 2035, and 2050. Statistics Norway’s main alternative was used in the projections. Fourth, we performed weighted analyses to estimate the potential reduction in number of people with depression in the future in scenarios where the prevalence of loneliness at HUNT4 (and in the future) was reduced by 10%, 20%, 30%, 40%, 50%, or completely abolished (100% reduction). This calculation assumes a causal relationship between loneliness and depression, as found in the review study by Van As. The calculation can be debatable, as it was not found by the present study, but still, such a calculation could be useful as it will estimate the potential. If the assumption fails, or there is only a weak causal relationship, changing the prevalence of loneliness would have smaller impact on depression prevalence than in our estimates. The weights were constructed as follows: In HUNT4, 2669 respondents reported loneliness, and 7563 respondents reported no loneliness. In the scenario where loneliness was to be reduced by 10%, the number reporting loneliness would have to be reduced to 2402 (2669–2669*0.10), and the group without loneliness would increase correspondingly to 7830 (7563 + 2669*0.10). Translated into weights, those with loneliness were given a deflated weight of 0.9 (2402/2669), and those without loneliness were inflated by the weight of 1.04 (7830/7563). We applied corresponding weights for the other scenarios. We applied weighted binominal regression analysis with an identity link to test differences between groups, and predictions from the regression model were performed to estimate depression prevalence with accompanying 95% confidence intervals at fixed values for the covariates in the regression model. These predictions were also used to calculate average marginal effects, to test for differences in depression prevalence between specific set of covariates, for example between men and women at same age and living situation. In the margins command, the Delta-method was applied with an accompanying t-test for estimation of significance and 95% confidence intervals. In a sensitivity analysis to consider that some respondents participated in more than one of the surveys with repeated measurements of loneliness, we applied cluster-robust variance estimates using the vce cluster command in Stata, relaxing the usual requirement that the observations must be independent. This option will produce unbiased standard errors even if the observations are correlated, which would typically be the case in settings where we have repeated measurements within individuals, as in our setting. However, prevalence results were identical to three decimal places, so we did not use this correction in the main analyses as the weighting procedure did not accommodate for the vce cluster approach. Fifth, as we wanted to investigate predictors of depression, we performed a series of age- and sex-adjusted logistic regression models with depression as the dependent variable, and the variables HUNT wave, living situation, education, and loneliness added one by one in models 1–6, and all variables except loneliness added simultaneously in model 5, and finally all variables in model 6. Logistic regression was used for this purpose due to non-convergence for the binominal regression. Statistical significance was set to 5%.

### Ethical considerations

The study is part of the project “Years at healthy functioning among older adults in Norway,” which meets the guidelines for protection of human data concerning safety and privacy at the Norwegian Institute of Public Health, Oslo. It was approved by the Norwegian Regional Ethics Committee for medical research (REC), reference number 2019/149, Norway. Participants in the HUNT surveys gave written informed consent. The project “Years at healthy functioning among older adults in Norway”, leaded by the present manuscript’s last author, Bjørn Heine Strand, received funding from Helsedirektoratet (The Norwegian Directorate of Health). The funders had no role in study design, data collection and analysis, decision to publish, or preparation of the manuscript.

## Results

### Sample characteristics

Mean age was 76.6 years (SD = 5.0) at HUNT2, 77.0 years (SD = 5.2) at HUNT3, and 76.9 years (SD = 5.6) at HUNT4. There were more women than men in the study: 55.5% women in HUNT2, 55.2% in HUNT3, and 53.4% in HUNT4. The prevalence of individuals living alone decreased significantly from 37.4% in HUNT2 to 35.1% in HUNT3 and 32.1% in HUNT4. Loneliness decreased over time; at HUNT2, 11.2% often felt lonely, while at HUNT4, the prevalence was 5.1%. Table 1 shows demographic characteristics among those with and without depression.

**Table 1:** Sample characteristics among older adults with and without depression (HADS-D>=8) in HUNT2-4. N = 26,512. The HUNT Study, Norway.

	HUNT surveys											
	HUNT2				HUNT3				HUNT4			
	HADS-D>=8				HADS-D>=8				HADS-D>=8			
	No, n (%)		Yes, n (%)		No, n (%)		Yes, n (%)		No, n (%)		Yes, n (%)	
Sex												
Women	4406	(81.9)	975	(18.1)	3460	(86.3)	551	(13.7)	4611	(90.3)	493	(9.7)
Men	3504	(81.3)	804	(18.7)	2749	(84.6)	500	(15.4)	3903	(87.5)	556	(12.5)
Age												
70-74	3309	(83.9)	633	(16.1)	2470	(88.4)	324	(11.6)	3708	(91.4)	351	(8.6)
75-79	2596	(80.7)	619	(19.3)	1933	(84.9)	344	(15.1)	2593	(91.0)	258	(9.0)
80-84	1356	(79.1)	359	(20.9)	1274	(84.8)	229	(15.2)	1354	(85.4)	232	(14.6)
85+	649	(79.4)	168	(20.6)	532	(77.6)	154	(22.4)	859	(80.5)	208	(19.5)
Education												
Primary	5961	(79.9)	1497	(20.1)	3755	(84.0)	715	(16.0)	3614	(87.1)	534	(12.9)
Secondary	1343	(87.5)	191	(12.5)	1627	(86.9)	245	(13.1)	3137	(89.4)	372	(10.6)
Tertiary	426	(88.6)	55	(11.4)	646	(90.9)	65	(9.1)	1755	(92.7)	139	(7.3)
Lives alone												
No	4408	(82.7)	925	(17.3)	4033	(86.1)	653	(13.9)	5839	(90.4)	620	(9.6)
Yes	2595	(81.3)	595	(18.7)	2145	(84.6)	390	(15.4)	2636	(86.5)	413	(13.5)
Lonely												
Never	4678	(88.0)	640	(12.0)	4196	(90.0)	464	(10.0)	5990	(93.0)	451	(7.0)
Sometimes	1268	(74.2)	442	(25.8)	964	(78.5)	264	(21.5)	1374	(81.2)	319	(18.8)
Often	540	(61.0)	345	(39.0)	272	(64.0)	153	(36.0)	293	(66.4)	148	(33.6)

HADS-D: Hospital Anxiety and Depression Scale. HUNT: The Trøndelag Health Study.

**Fig 1:**
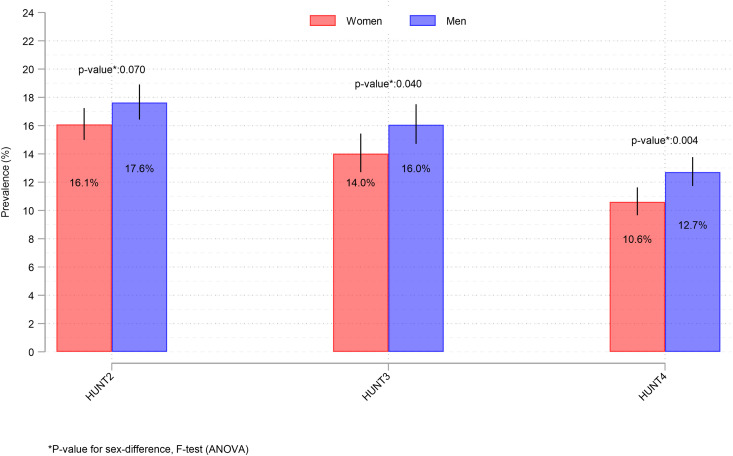
Standardized** prevalence of depression (HADS-D>=8) with 95% CI by sex and year, age 70 + . Text below figure: ** Standardized prevalence was estimated using standardization weights, where weights were based on the Norwegian population using three sets of Norwegian standard populations by age (70-74, 75-79, 80-84, 85+), education (primary, secondary, tertiary), and sex (men, women), for the years 1995 (for HUNT2), 2006 (for HUNT3), and 2016 (for HUNT4). Weights were supplied from the National database microdata.no. The purpose of the standardization was to get national estimates and to correct for lower participation rates in the higher age groups, among men, and in the lower education groups. [Stata code: svy, subpop(HUNT2): proportion depression, over(sex). This was run 3 times, one for each HUNT survey]. Testing for differences in depression between men and women was done using the Fisher-test adjusted by the survey design by using the contrast command in Stata after the command above (contrast r.sex@1.depression).

Standardized prevalence of depression declined significantly over time in both men and women (Fig 1). For women, the standardized prevalences at HUNT2, HUNT3, and HUNT4 were 16.1% (95% confidence interval (CI) 15.0, 17.2), 14.0% (12.7, 15.4), and 10.6% (9.7, 11.6), respectively. For men, the corresponding numbers were 17.6% (16.4, 18.9), 16.1% (14.7, 17.5), and 12.7% (11.7, 13.8). In women, the prevalence was significantly lower at HUNT3 compared with HUNT2 (F(1,27950), F = 5.24,: p = 0.022), as well as lower at HUNT4 compared with HUNT3 (F(1,27950), F = 15.74, p < 0.001). In men, the prevalence was not significantly lower at HUNT3 compared with HUNT2 (F(1, 28384), F = 2.71, p = 0.099), but it was significantly lower at HUNT4 compared with HUNT3 (F(1,28384), F = 14.32, p < 0.001) and HUNT2 (F(1, 28384): F = 36.00, p < 0.001). Men had a significantly higher standardized prevalence of depression than women at HUNT3 (F(1,29189, F = 4.20, p = 0.04)) and HUNT4 (F(1,28255, F = 8.49, p = 0.004)), but not at HUNT2 (F(1,29148, F = 3.28, p = 0.07)) (Fig 1).

**Fig 2:**
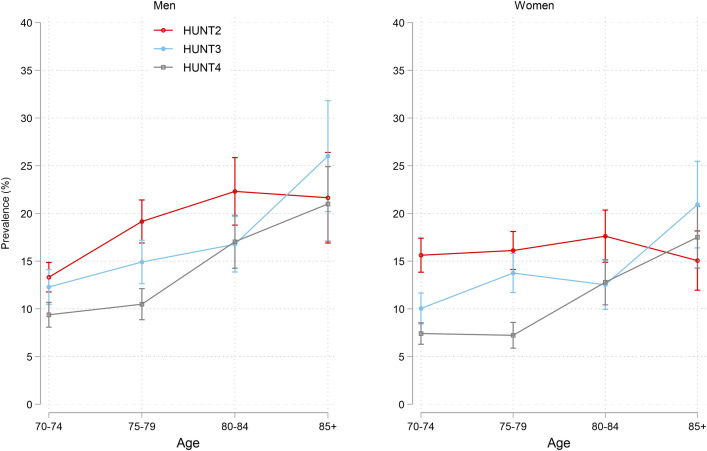
Standardized prevalence of depression (HADS-D>=8 with 95% CI) by sex, time, and age group. Text below figure: * Standardized prevalence was predicted from a weighted regression model. [The stata code: svy: glm depression survey##sex##age, family(bin) link(identity)]. The weights used for standardization were based on the Norwegian population using three sets of Norwegian standard populations by age (70-74, 75-79, 80-84, 85+), education (primary, secondary, tertiary), and sex (men, women), for the years 1995 (for HUNT2), 2006 (for HUNT3), and 2016 (for HUNT4). Weights were supplied from the National data base microdata.no. The purpose of the standardization was to get national estimates and to correct for lower participation rates in the higher age groups, among men, and in the lower education groups.}. Average marginal effects were predicted using the margins command, and differences between average marginal effects were estimated applying the dydx command, which applies the Delta method and t-test.

In men, there was an age gradient with higher depression prevalence at higher ages at all study waves, while in women, such age gradients were only present at HUNT2 and HUNT4 (Fig 2). At HUNT4, the standardized prevalence of depression in men was 21.0% in the 85 + year-olds (95% CI 17.1, 24.9) as compared to 9.4% among the 70–74-year-olds (95% CI 8.1, 10.7). Similarly, in women at HUNT4, the prevalence was 17.5% in the 85 + year-olds (95% CI 14.3, 20.7%), compared to 7.4% among the 70–74-year-olds (95% CI 6.3, 8.5).

**Fig 3:**
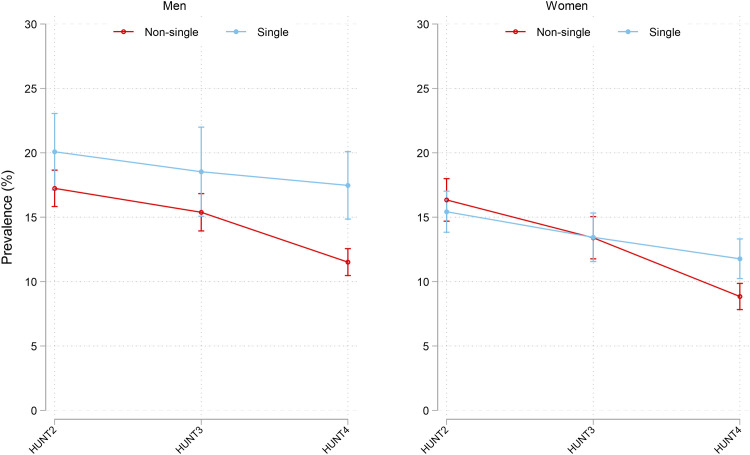
Standardized prevalence* HADS-D>=8 with 95% CI by sex, time, age group, and living situation. N = 22801. Text below figure: * Standardized prevalence was predicted from a weighted regression model adjusted by age. [The stata code: svy: glm depression survey##household##sex age, family(bin) link(identity)]. The weights used for standardization were based on the Norwegian population using three sets of Norwegian standard populations by age (70-74, 75-79, 80-84, 85+), education (primary, secondary, tertiary), and sex (men, women), for the years 1995 (for HUNT2), 2006 (for HUNT3), and 2016 (for HUNT4). Weights were supplied from the National data base microdata.no. The purpose of the standardization was to get national estimates and to correct for lower participation rates in the higher age groups, among men, and in the lower education groups. Average marginal effects were predicted using the margins command, and differences between average marginal effects were estimated applying the dydx command, which applies the Delta method and t-test.

At HUNT4, living alone was associated with higher standardized depression prevalence (F(1,28201, F = 35.29, p < 0.001), while at HUNT2 and HUNT3 there were no such differences by household size. For men, single household was associated with a 5.7 percentage points (pp) higher depression prevalence compared to those living with someone (pp 95% CI 2.9, 8.4) in a model adjusted by age (Fig 3). There were similar results for women; at HUNT4, living alone was associated with a 3.3 pp higher depression prevalence compared to those living together with someone (pp 95% CI 1.4, 5.3) in a model adjusted by age. Earlier study waves revealed no such significant differences by household type in men or women.

**Fig 4:**
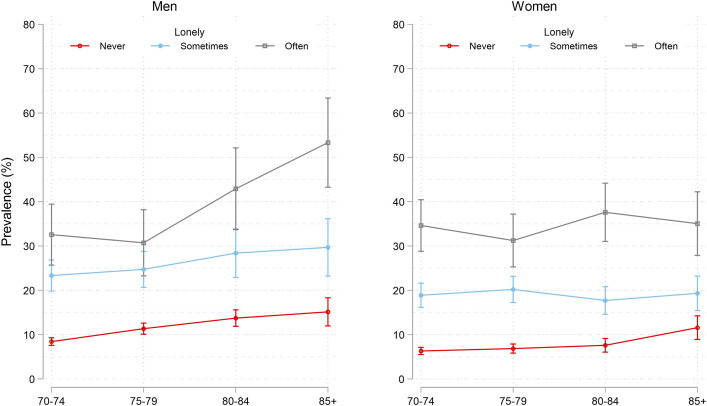
Standardized prevalence* of depression (HADS-D>=8) with 95% CI by sex, age, and self-reported loneliness. Text below figure: * Standardized prevalence was predicted from a regression model adjusted by survey wave. [The stata code: svy: glm depression loneliness##age##sex study, family(bin) link(identity)]. The weights used for standardization were based on the Norwegian population using three sets of Norwegian standard populations by age (70-74, 75-79, 80-84, 85+), education (primary, secondary, tertiary), and sex (men, women), for the years 1995 (for HUNT2), 2006 (for HUNT3), and 2016 (for HUNT4). Weights were supplied from the National data base microdata.no. The purpose of the standardization was to get national estimates and to correct for lower participation rates in the higher age groups, among men, and in the lower education groups. Average marginal effects were predicted using the margins command, and differences between average marginal effects were estimated applying the dydx command, which applies the Delta method and t-test.

Loneliness was associated with depression across men and women of all ages (Fig 4). Among men, depression rates were particularly high among those who were “often” lonely, in particular in the 80–84 (42.9%) and over 85 (53.3%) age groups, compared to younger age groups (70–74: 32.6% and 75–79: 30.7%). The rates among women were also high among those who were “often” lonely, but there was less variation across age groups (31.2–35.0%). In both men and women, rates were far lower in the “never lonely” group (Fig 4).

In a multiple logistic regression model adjusted by age and sex, candidate predictor variables were added one by one. The analysis revealed that the following factors were significantly associated with higher depression rates: early study wave, low education, loneliness, and living alone (Table 2). Living alone was not associated with higher odds for depression in the fully adjusted model 6. The association between living alone and depression was mediated by loneliness (see model 5 vs. model 6). There was no significant interaction between household status and loneliness (Table 2).

**Table 2:** Odds ratio (OR) for depression (HADS-D>=8) with 95% confidence interval (CI). Analyses are run using the weighted survey design on the sub-sample with non-missing for all included variables in model 6 (complete case analysis). Number of observations: N = 21,484 for all models 1-6, corresponding to the larger population size of N = 1,566,094.

Variable	Model 1	Model 2	Model 3	Model 4	Model 5	Model 6
	OR (95% CI)	OR (95% CI)	OR (95% CI)	OR (95% CI)	OR (95% CI)	OR (95% CI)
Sex						
Women	Ref	Ref	Ref	Ref	Ref	Ref
Men	1.23 (1.12, 1.34)	1.28 (1.17, 1.40)	1.49 (1.36, 1.63)	1.31 (1.19, 1.44)	1.37 (1.25, 1.51)	1.43 (1.30, 1.58)
Age						
70-74	Ref	Ref	Ref	Ref	Ref	Ref
75-79	1.20 (1.08, 1.33)	1.18 (1.07, 1.31)	1.15 (1.04, 1.28)	1.19 (1.08, 1.32)	1.15 (1.04, 1.28)	1.15 (1.04, 1.28)
80-84	1.51 (1.34, 1.70)	1.45 (1.29, 1.64)	1.29 (1.14, 1.46)	1.46 (1.29, 1.64)	1.40 (1.24, 1.58)	1.30 (1.15, 1.48)
85+	2.01 (1.76, 2.31)	1.87 (1.63, 2.16)	1.46 (1.26, 1.70)	1.80 (1.56, 2.08)	1.78 (1.54, 2.06)	1.60 (1.38, 1.86)
Education						
Primary		Ref			Ref	Ref
Secondary		0.67 (0.60, 0.74)			0.70 (0.63, 0.78)	0.72 (0.65, 0.81)
Tertiary		0.49 (0.42, 0.58)			0.54 (0.46, 0.64)	0.55 (0.47, 0.65)
Lives alone						
No				Ref	Ref	Ref
Yes				1.28 (1.16, 1.42)	1.23 (1.11, 1.36)	0.63 (0.56, 0.71)
Lonely						
Never			Ref			Ref
Sometimes			2.76 (2.48, 3.08)			3.30 (2.93, 3.72)
Often			5.57 (4.84, 6.40)			6.62 (5.67, 7.74)
Survey						
HUNT2	Ref	Ref			Ref	Ref
HUNT3	0.85 (0.76, 0,95)	0.85 (0.76, 0.95)			0.90 (0.81, 1.00)	0.97 (0.87, 1.09)
HUNT4	0.65 (0.59, 0.72)	0.65 (0.59, 0.72)			0.74 (0.66, 0.82)	0.79 (0.70, 0.88)

Included covariates: Model 1: Age, sex, and HUNT survey; Model 2: Age, sex, education, and HUNT survey; Model 3: Age, sex, and loneliness; Model 4: Age, sex, and household; Model 5: Age, sex, education, household, and HUNT survey; Model 6: Fully adjusted.

### Projections of depression in the future

Due to the aging population, based on fixed age- and sex-specific prevalence estimates at HUNT4, the projected number of persons aged 70 + years with depression will almost double by 2050 (Table 3). The prevalence of depression will increase from 12% in 2023 to 13% in 2050 and 15% in 2100.

**Table 3:** Projections of total number of 70 + yr olds with depression (HADS-D>=8) in Norway for years 2025, 2030, 2035, 2040, 2045, 2050, and 2100.

70+	est2023	est2025	est2030	est2035	est2040	est2045	est2050	est2100
Total, N	724 426	765 061	871 291	975 321	1 090 119	1 188 222	1 246 676	1646927
HADS-D>=8, n	84 146	89 609	105 675	121 768	137 635	151 971	163 866	239 317
%	12%	12%	12%	12%	13%	13%	13%	15%
SE	2 449	2 612	3 136	3 722	4 261	4 780	5 306	9 170
95% CI lower	79 345	84 489	99 528	114 473	129 284	142 602	153 466	221 344
95% CI upper	88 947	94 729	111 822	129 063	145 986	161 340	174 266	257 290

Method: Based on HUNT4 estimates of depression prevalence projected on the projected number of 70 + yr olds in the coming decades using population projections from Statistics Norway (table 13599: Population projection January 1st by sex and age. Main alternative).

### Potential reduction in depression by reduction in loneliness

Assuming there is a causal relationship between loneliness and depression of strength observed in the current study, and further assuming, in a counterfactual scenario, that loneliness in the 70 + population was reduced by 10%, the depression prevalence in this age group would be reduced from 12% to 11%. In the scenario where loneliness was removed entirely, depression would further decline to 8% (Table 4). These estimates assumed that there is a causal relation between loneliness and depression, although, based on our data, we cannot conclude that there is such a relation.

**Table 4:** Potential reduction in number (and %) with depression in the Norwegian 70 + population for year 2023 in the scenarios where loneliness is reduced by 10%, 20%, 30%, 40%, 50%, and 100%.

Reduction in loneliness, %	Number with depression	Depression prevalence (%)
0% (actual estimate for year 2023)	84146	12%
10%	81743	11%
20%	79489	11%
30%	77064	11%
40%	74755	10%
50%	72297	10%
100%	59813	8%

## Discussion

In the present study, we found a substantial decrease in the prevalence of depression among older Norwegians over the last 20 years. However, this was not the case for the oldest adults, those over 85. Furthermore, men had higher depression rates than women in the most recent surveys. Low education and loneliness were also associated with depression. The number of older adults with depression is expected to almost double by 2050. However, if loneliness could be reduced by 50%, and assuming that there Is a causal relationship between loneliness and depression, the number of older adults with depression could potentially be reduced by 14%.

Depression declined in men and women 70+ during 1995–2019, from 16.1% to 10.6% in women and from 17.6% to 12.7% in men. The rate in the last wave is in the lower range compared to a review study reporting an average prevalence of 13.3% among older adults [9], but comparable to a study indicating that Scandinavian countries had the lowest rates [29]. Our findings are also consistent with results from another Norwegian population study among older adults using HADS-D as a marker for depression [15] and data from HUNT covering the 70–79 age group [14]. These decreasing rates in Norwegian studies might be due to the high educational levels, generous welfare provisions, and low levels of poverty in Norway, as low socioeconomic levels and low income are well-known risk factors for depression [8]. Indeed, the present study found that low education was also associated with depression in the population 70 + .

Previous studies from HUNT have shown that the older Norwegian population has become both healthier and better functioning over time [30], as well as less lonely [31]. This might partly explain why the rates of depression have decreased over the years. The present study has also shown that there are fewer older adults living alone, which might also have contributed to the decrease in depression.

Older men had higher levels of depression than older women in the two most recent waves. This is contrary to previous studies reporting higher rates of depression among females [8], but consistent with findings from another Norwegian population study [15]. However, another study found an increase in depressive symptoms over time among men over 70 years of age, but not women [16). Higher rates of suicide among elderly men are reported [32], which might indicate that men report depressive symptoms to a lesser extent than women and are less amenable to health care. Furthermore, there is an ongoing discussion regarding the phenotype of depression and how it is measured by HADS-D, as this scale lacks somatic symptoms of depression, such as loss of appetite and loss of sleep, focusing mostly on symptoms that measure anhedonia. This might contribute to an absent or reversed sex difference [17].

A robust association was found between higher age and depression rates in both men and women, affirming the trend of increased rates among older adults [8,9,15,33–36].

Our discovery that the connection between depression and living alone is influenced by loneliness aligns with research indicating that living alone can contribute to feelings of loneliness. However, it is important to note that not all elderly individuals who live alone experience loneliness [3]; living alone is not a risk factor for depression in itself, but feeling lonely is [37]. This has also been shown in another study that found that subjective rather than objective quality of social interactions predicted depressive symptoms, suggesting that the perceived quality of the social interaction rather than its frequency is of importance [38].

The present study has limitations, which might affect our results. Firstly, we used a depression scale to determine depression prevalence. Even a validated depression scale, such as HADS, only indicates the presence of the level of depressive symptoms and does not serve as a diagnostic tool. Thus, our findings cannot establish true depression prevalence rates, as is the case for most large population studies. There is also evidence that HADS has lower sensitivity and specificity than other instruments for depression, even though the cut-off we used is the recommended for a good balance between sensitivity and specificity, but with a slightly higher specificity according to a meta-analysis [19]. Another limitation of the HADS-D is that it does not include somatic symptoms, such as loss of appetite and loss of sleep, which could have led to underestimation of depression in this group. Furthermore, there is limited evidence regarding the use of HADS among older adults above 80 years [39]. Secondly, we did not include health status, a well-known correlate of depression, as an independent factor in the multivariate analysis. Thirdly, the projections are uncertain and rely on a set of assumptions which might be unrealistic. For instance, fixing the standardized prevalence of reported loneliness in the last wave only, not considering the changes in loneliness prevalence in all study waves, implies that we do not expect loneliness rates to change in the future. Previous Norwegian studies have shown that the population has become better functioning over time. As worse functioning is a known risk factor for depression, it might be that the prevalence of depression decreases even more. Nevertheless, we believe the estimates could be of public health relevance. Fourthly, we used a crude one-item tool to measure loneliness, whereas multidimensional tools are common in current research practice [40]. Finally, the participation rates decreased over time, especially from HUNT2 to HUNT3 (23), which might have increased healthy selection bias in the last waves. However, we believe this to be a minor issue, as the last survey captured more frail individuals by allowing home-based interviews. Our standardization of the prevalence also takes into account differences in the waves regarding age, sex, and education. This study has the strength of including a large representative Norwegian sample of more than 20,000 older adults over a long period of 20 years.

## Conclusion

In conclusion, the present study revealed a decline in depression rates over time, yet a consistent high prevalence persisted among the oldest old. Notably, men aged 85 and above who reported experiencing loneliness were the group with the highest depression prevalence, exceeding a noteworthy 50%. This stands in contrast to their counterparts of the same age who did not report loneliness, exhibiting a markedly lower prevalence of 13%. The robust correlation observed between loneliness and depression underscores an opportunity for prevention. Effectively addressing and mitigating loneliness has the potential to reduce depression rates, offering substantial benefits to both community well-being and the public health care system.
